# Complex imaging of phase domains by deep neural networks

**DOI:** 10.1107/S2052252520013780

**Published:** 2021-01-01

**Authors:** Longlong Wu, Pavol Juhas, Shinjae Yoo, Ian Robinson

**Affiliations:** a Computational Science Initiative, Brookhaven National Laboratory, Upton, NY 11973, USA; b Condensed Matter Physics and Materials Science Department, Brookhaven National Laboratory, Upton, NY 11973, USA; c London Centre for Nanotechnology, University College London, London, WC1E 6BT, United Kingdom

**Keywords:** machine learning, Bragg coherent X-ray diffraction, phase retrieval, single-particle imaging, deep neural networks

## Abstract

The reconstruction of a single-particle image from the modulus of its Fourier transform, by phase-retrieval methods, has been extensively applied in X-ray structural science. Particularly for strong-phase objects, such as the phase domains found inside crystals by Bragg coherent diffraction imaging (BCDI), conventional iteration methods are time consuming and sensitive to their initial guess because of their iterative nature. Here, a deep-neural-network model is presented which gives a fast and accurate estimate of the complex single-particle image in the form of a universal approximator learned from synthetic data. A way to combine the deep-neural-network model with conventional iterative methods is then presented to refine the accuracy of the reconstructed results from the proposed deep-neural-network model. Improved convergence is also demonstrated with experimental BCDI data.

## Abstract 2

Machine-learning approaches can greatly facilitate single-particle-imaging experiments at X-ray free-electron-laser facilities by providing real-time images from the coherent X-ray diffraction data stream, using methods presented in this article.

## 1. Introduction   

Single-particle imaging by using coherent X-ray diffraction was proposed more than a decade ago by the work of Fienup (1978 ▸), Miao *et al.* (1999 ▸), Robinson *et al.* (2001 ▸), Chao *et al.* (2005 ▸) and Sakdinawat & Attwood (2010 ▸). As a method of determining the inside complex structure of an individual particle, it records the diffracted coherent X-ray intensity by the particle in reciprocal space, where the phase information of the corresponding intensity is lost during the measurement (Williams *et al.*, 2003 ▸; Chapman *et al.*, 2006 ▸; Pfeifer *et al.*, 2006 ▸). To provide this missing phase, one crucial step in an X-ray single-particle-imaging experiment, either by forward-scattering X-ray coherent diffraction imaging (Xu *et al.*, 2014 ▸) or Bragg coherent diffraction imaging (BCDI) (Newton *et al.*, 2010 ▸; Yang *et al.*, 2013 ▸), is the reconstruction of the real-space complex information of the particle from its measured X-ray diffraction-pattern intensity. Because of the loss of the phase information of the recorded X-ray intensity, iterative phase-retrieval algorithms are widely applied to reconstruct the complex structure information of the measured particle. As shown originally by Bates (1982 ▸), this process, known as phase retrieval, depends on the diffraction data being oversampled by at least a factor of two with respect to the Shannon–Nyquist frequency. Except for pathologically constructed special cases, a unique reconstruction result is expected in two or three dimensions, subject to the known symmetries of the Fourier transform.

In last few decades, many phase-retrieval algorithms have been developed, such as error reduction by Gerchberg & Saxton (1972 ▸), hybrid input–output by Fienup (1982 ▸), difference map by Elser (2003 ▸) and the relaxed averaged alternating reflection by Luke (2005 ▸). These are commonly utilized and have found successful application in the coherent X-ray diffraction community. These phase-retrieval methods usually require hundreds or thousands of iterations to converge to a solution with high confidence, which is exceptionally time consuming and sensitive to the initiating guess of the phase of the X-ray intensity, the assumed ‘support’ boundary of the measured particle, as well as the choice of algorithms. Marchesini *et al.* (2003 ▸), Huang *et al.* (2010 ▸) and Wang *et al.* (2020 ▸) have found that it is crucial to find a correct support for the convergence of these methods because the particle shape is generally hard to determine directly from the measured coherent X-ray diffraction data. Marchesini *et al.* (2003 ▸) proposed a ‘shrink wrap’ support method and Wang *et al.* (2020 ▸) described an averaged support method to dynamically modify the support by adapting it to the shape of the particle, but these still require a certain number of iterations to converge. Besides, there is strong scientific interest in studying structured nanoparticles containing phase-shifted structural domains which are relevant to their function as superconductors, catalysts or piezoelectric actuators, for example. For these ‘strong phase’ particles, which have multicentered diffraction patterns, the iterative method struggles to obtain a correct solution with high confidence (Ihli *et al.*, 2016 ▸; Robinson *et al.*, 2020 ▸). Consequently, the difficulty of coherent diffraction imaging to deliver trustworthy unique solutions to the phase problem currently limits the range of applications of the iterative phase-retrieval method in single-particle-imaging experiments. With the advent of ultrafast X-ray free-electron lasers (XFELs), time-resolved single-particle-imaging experiments are more and more addressing ultrafast phenomena in physical or chemical processes within nanoparticles, such as melting, driven fluctuations, *etc*. (Clark *et al.*, 2013 ▸; Gomez *et al.*, 2014 ▸; Rupp *et al.*, 2017 ▸; Rose *et al.*, 2018 ▸; Ihm *et al.*, 2019 ▸; Wen *et al.*, 2019 ▸; Sobolev *et al.*, 2020 ▸; Mudrich *et al.*, 2020 ▸). These experiments would benefit enormously from the development of a fast and reliable phase-retrieval method to reconstruct live images of particles in real time during the execution of these time-resolved experiments.

Deep-neural-network-based machine-learning methods have been considered as a revolution for the reconstruction algorithm. The convolutional neural network (CNN) has seen remarkable progress for image synthesis with several recent successful applications by Ronneberger *et al.*, (2015 ▸), Nguyen *et al.*, (2018 ▸), Cherukara *et al.* (2018 ▸) and Guan & Tsai (2019 ▸). The CNN is a non-iterative end-to-end method. Once a trained model has been obtained, the phase information of the measured particle can be recovered from the coherent X-ray diffraction patterns in milliseconds, which is therefore suitable for ultrafast coherent X-ray diffraction experiments. Cherukara *et al.* (2018 ▸) presented the idea that two parallel trained models can separately reconstruct the phase and amplitude of a complex object from its coherent X-ray diffraction intensity. However, since CNN methods are described as universal approximators learned from data, they cannot be quantitatively accurate for all samples and they are prone to shifts based on the distribution of the training data. Especially when working with data from a new experiment, this shift is inevitable since the measured data are unknown to the model. Therefore, the accuracy of the reconstructed complex information from deep-neural-network methods still needs to be refined.

In this work, we developed a CNN model to reconstruct a complex image, containing both amplitude and phase information, of a particle in real space from the modulus of its Fourier transform in reciprocal space. By using a comprehensive model, we are able to recover the amplitude and phase information of the particle at the same time. Our developed CNN model can provide a very high accuracy reconstruction at a speed which is much faster than the conventional iterative phase-retrieval methods. The developed method will be very suitable for a real-time single-particle-imaging experiment, especially for ultrafast X-ray single-particle experiments, where the current iterative phase-retrieval methods are hard to apply. Furthermore, we also present a way to combine our CNN model with the conventional iteration method to refine the accuracy. We show that the machine-learning CNN approach gets much closer to the correct solution than a nearest-neighbour search (NNS) of the same set of reference diffraction patterns used for training. The outcomes from the CNN model, the amplitude, phase and support (obtained after the binarization of the predicted amplitude), are then fed as an initiation guess into the conventional iteration methods, which shows better convergence and a more accurate result.

## 2. Results   

### 2.1. CNN model training and testing   

**Figure 1:**
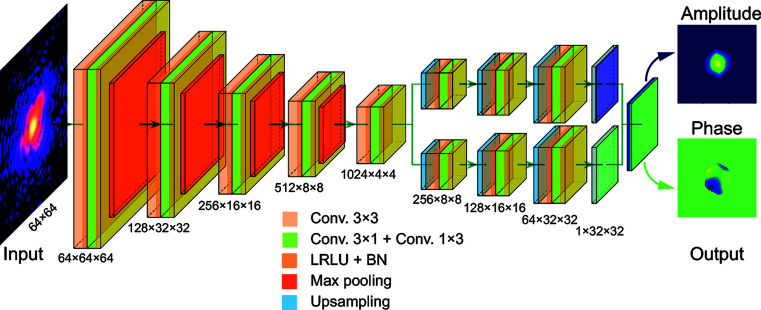
A schematic visualization of the deep neural network for single-particle-imaging inversion. The neural network is implemented using an architecture composed entirely of convolutional, maximum pooling and upsampling layers. In the network, Conv. refers to convolution, LRLU refers to the leaky rectified linear unit and BN refers to batch normalization. There are two channels in the final output layer, for the reconstruction of the amplitude and the phase of the particle. All activations are related to the LRLU, except for the final convolutional layer which uses RLU activations.

Our developed machine-learning model is based on a CNN framework that adopts the general ‘encoder–decoder’ architecture, as presented in Fig. 1 ▸. It consists of two connected convolutional blocks to separately output the amplitude and phase information of a particle from its coherent X-ray diffraction data. The model mainly has three parts. The first part is a convolutional autoencoder, which represents the underlying manifold of the input coherent X-ray diffraction data in feature space (Ronneberger *et al.*, 2015 ▸; Cherukara *et al.*, 2018 ▸). Then, the encoded result is equally divided into two independent deconvolutional decoder parts to generate the amplitude and phase information of the measured particle. For the proposed model, we used the leaky rectified linear unit (LRLU) for all activations, except for the final convolutional layer where the rectified linear unit (RLU) was used. The modules shown in Fig. 1 ▸ to connect the input to the output are one type of convolution block (3 × 3 convolution + LRLU + BN, where BN refers to batch normalization), followed by another type of convolution block (3 × 1 convolution + 1 × 3 convolution + LRLU + BN). Additionally, it should also be mentioned that the size of the output amplitude or phase is one quarter of the size of the input diffraction pattern in our model (*i.e.* the size of the output amplitude or phase at each dimension is half of the size of the input diffraction, as shown in Fig. 1 ▸), in the spirit of keeping the problem overdetermined.

**Figure 2:**
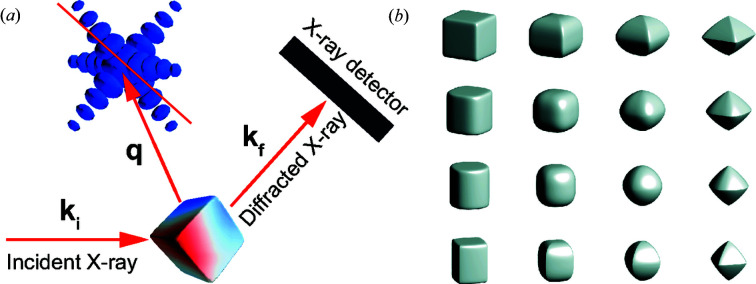
Construction of the single-particle coherent X-ray diffraction training data. (*a*) A schematic illustration of a typical coherent X-ray diffraction experimental setup. (*b*) Representative particle shapes produced by the superellipsoid function described in the text.

Since the goal of any single-particle coherent X-ray diffraction imaging experiment is to numerically compute the complex-valued information inside a particle from the obtained coherent X-ray diffraction pattern, different variants of coherent X-ray diffraction experiments interpret this complex information in different ways. For coherent-diffraction-imaging experiments, Chapman *et al.* (2006 ▸) represented the reconstructed information as the local complex refractive index of a particle. In BCDI experiments, Robinson *et al.* (2001 ▸) and Williams *et al.* (2003 ▸) identified the phase of the reconstructed information as the local crystal lattice strain inside a particle via the Bragg diffraction geometry, plus a small contribution from refraction in the crystal discovered by Harder *et al.* (2007 ▸). In all cases, the coherent X-ray diffraction intensity *I*(**Q**) measured in these variants of single-particle experiments is given by the modulus squared of the Fourier transform of this corresponding complex field:
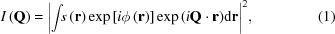
where **Q** = **q** − **h**, **q** = **k**_f_ − **k**_i_ is the momentum transfer defined by the incident X-ray wavevector **k**_i_ and the diffracted X-ray wavevector **k**_f_, and **h** is a reciprocal lattice vector of the crystal. Here, 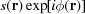 represents the complex-valued information inside the particle, where *s*(**r**) and ϕ(**r**) are the amplitude and phase distribution of the particle, respectively. Usually, *s*(**r**) is the shape function of the particle with *s*(**r**) = 0 outside and *s*(**r**) = 1 inside the particle. It can be seen that both the coherent X-ray diffraction intensity *I*(**Q**) and the particle density are in 3D space. Fig. 2 ▸(*a*) shows a schematic illustration of the diffraction geometry of a typical single-particle-imaging experiment. The 3D diffraction intensity in reciprocal space is usually recorded by a 2D detector. The recorded intensity is a slice of the 3D diffraction intensity, where the slice plane is determined by the experimental geometry, as shown in Fig. 2 ▸(*a*). Especially for an ultrafast X-ray experiment, only the 2D coherent X-ray intensity slice through the centre of the peak is recorded. Thus, to investigate the performance of our proposed CNN model, complex-valued real-space particles are needed to obtain the 2D coherent X-ray diffraction patterns. Generally, for a particle with an anisotropic shape *s*(**r**), its shape can be introduced into the formalism by applying an analytic or numerical computation. For demonstration purposes, we consider a shape known as a superellipsoid (Gridgeman, 1970 ▸; Wriedt, 2002 ▸), whose implicit form is written as
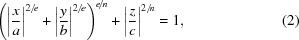
where *a, b*, and *c* define the bounds along the *x*, *y* and *z* directions, respectively. Exponents *n* and *e* are the roundedness parameters. As illustrated in Fig. 2 ▸(*b*), these parameters, *a, b, c, e*, and *n*, allow one to continuously and widely vary the particle shape. Specifically, *a* = *b* = *c*, *e* = 1 and *n* = 1 yields a sphere, while *a* = *b* = *c*, *e* = 2 and *n* = 2 describes an octahedral shape. Generally, since the phase information of real-world particles is diverse, it is hard to use one general function to describe all of them. We used a 3D Gaussian-correlated profile (Garcia & Stoll, 1984 ▸) to simulate the phase information ϕ(**r**) of the particles, which is given as
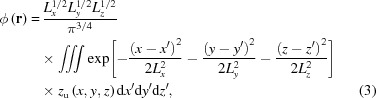
where *z*_u_(*x*, *y*, *z*) obeys an uncorrelated Gaussian random distribution. *L*_*x*_, *L*_*y*_ and *L*_*z*_ are the transverse correlation lengths along the *x*, *y* and *z* directions, respectively. Once described by equations (2) and (3), the complex field 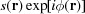 is randomly orientated into different directions by a 3D rotation matrix to obtain the 3D complex field of a particle. The phase inside the particle is allowed to span from −π to π to represent the ‘strong phase’ limit described above. Finally, the 2D diffraction intensity of the particle is obtained by taking the central slice of the 3D diffraction intensity, the phase is deleted and only the intensity information is retained. Additionally, a Gaussian filter is also applied to smooth the edges of the particle before the Fourier transformation.

By applying this method to a wide range of random parameters, we simulated 2D diffraction patterns to be used as the training data to train the proposed CNN model. In the results reported here, we generated 150 000 simulated diffraction patterns, and the CNN model was trained in a supervised fashion, with the output amplitude and phase of the particle being considered as known *a priori*. The phase of the particle was shifted and scaled to (0, 1), and the phase outside the particle is set to zero. During the training, the training data were split into two disjoint sets, where 95% of them were used as training data and 5% of them were kept for subsequent testing.

**Figure 3:**
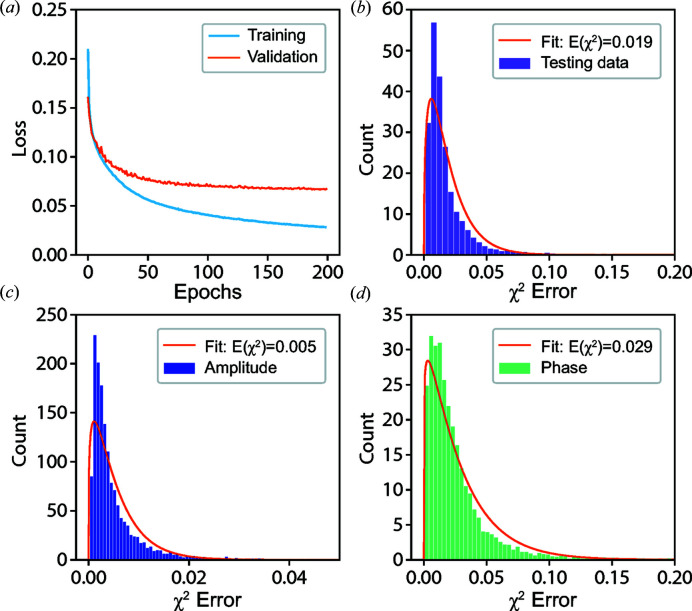
The performance of the CNN model and χ^2^ error in testing. (*a*) Training and validation loss as a function of the training epochs for the CNN model. (*b*) Histogram of χ^2^ error for the modulus of the coherent X-ray diffraction intensity and its corresponding fitting. Furthermore, histograms of χ^2^ error for the corresponding amplitude (*c*) and phase (*d*) of the particle in real space and their corresponding fitting results.

**Figure 4:**
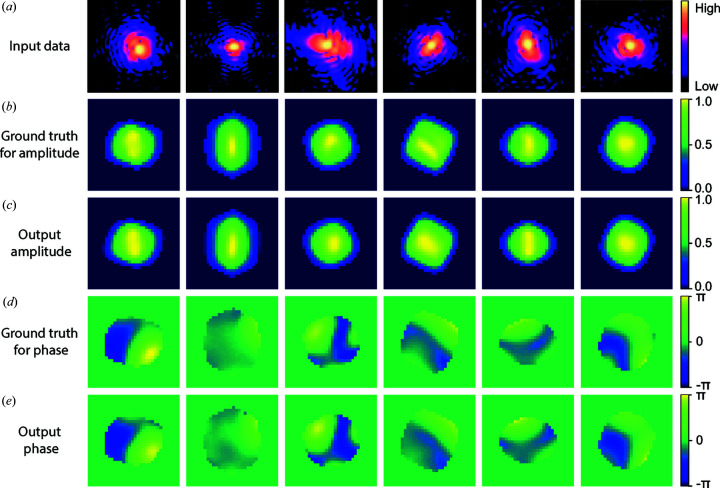
The representative results for the CNN model in testing. (*a*) Input testing coherent X-ray diffraction patterns. (*b*) The ground truth and (*c*) the corresponding predicted amplitude of the particles. (*d*) The ground-truth phase structure and (*e*) the corresponding predicted phase of the particles.

Fig. 3 ▸(*a*) shows the training and validation loss as a function of the training epochs. Each epoch refers to one complete pass of the training data. Since our proposed model can output the amplitude and phase of the particle at the same time, the loss (or error metric) for both training and validation is computed using a self-defined loss function (see Appendix *A* [App appa] for details), which is used to constrain their relation in real space as well as in reciprocal space at the same time. From Fig. 3 ▸(*a*), it can be seen that the training and validation loss are decreasing as the epoch is increasing. Even after training for more epochs, the validation loss is still decreasing. Since no divergence occurs, this indicates the stability of our CNN. Since the validation loss of our CNN model is computed by a self-defined loss function, we use the χ^2^ error (see Appendix *B* [App appb] for details) to estimate the quality of the reconstructed images in comparison with the ground truth of the testing data, which is commonly used in iterative phase-retrieval methods. Figs. 3 ▸(*b*)–3(*d*) present the histograms of the χ^2^ error for the modulus of the coherent X-ray intensity in reciprocal space, together with the amplitude and phase of the imaged particle in real space. The computed χ^2^ errors in the testing data lie in narrow ranges, which indicates that our CNN model shows excellent performance in reconstructing the complex image of a particle from its modulus in reciprocal space. Furthermore, by fitting the corresponding error with a Gamma distribution function, the average χ^2^ error for the modulus is 0.019, for the amplitude is 0.005 and for the phase is 0.029. Since the phase distribution of the particles is generally more complicated than their amplitude, it is expected that the error of the reconstructed phase is greater than that of the amplitude, as seen. The χ^2^ error of the modulus lies in the middle of the two. By varying the range of models used for training the proposed CNN model, we also noticed that the proposed model had better performance (smaller errors) when the phase range of the particles was made narrower. Fig. 4 ▸ shows six representative results of reconstructions from testing data not used for training. It can be seen that the current CNN model shows a remarkable performance on the reconstruction of the amplitude and phase information of a particle from its previously unseen coherent X-ray diffraction intensity pattern.

### 2.2. Comparison of CNN model with NNS and iteration methods   

The proposed CNN model is a machine-learning method of phase retrieval, which, once trained, provides a very fast (∼0.5 ms computation in our case) inversion of a diffraction pattern, unlike from an iterative phase-retrieval method. As we showed in Figs. 3 ▸ and 4 ▸, it can give an excellent reconstruction of testing data with very high accuracy. However, as a deep neural network, it works essentially as a deep approximator that learns from data chosen within a range of expected images. It is not expected that the proposed model would be quantitatively accurate for all coherent X-ray diffraction data, though it is very capable for a range of comprehensive complex-valued particles. When working with new data, it is prone to shifts based on the type of training dataset distributions. Also, an obvious question is whether the machine-learning approach provides any advantage over a NNS over calculated structures. We tested this by ‘look up’ of the best agreement of a new diffraction pattern with the 150 000 reference structures used for training. The average χ^2^ error over 300 ‘unseen’ test diffraction patterns was ∼0.08 for NNS compared with ∼0.02 for CNN for these same 300 diffraction patterns, The look-up procedure also took ∼30 s per pattern on our hardware, without any attempt at optimization, compared with ∼0.5 ms.

As we mentioned before, the other approach, iterative phase retrieval, is sensitive to the initial guess of the phase and the support. As reported in a number of articles, a good support is especially crucial for the iteration methods. On the other hand, the iterative methods are good at refining the output steadily if they are started under proper conditions. In this section, we demonstrate that using our proposed CNN model can provide a good initial guess for iterative algorithms to reconstruct the finer structural features of a particle.

**Figure 5:**
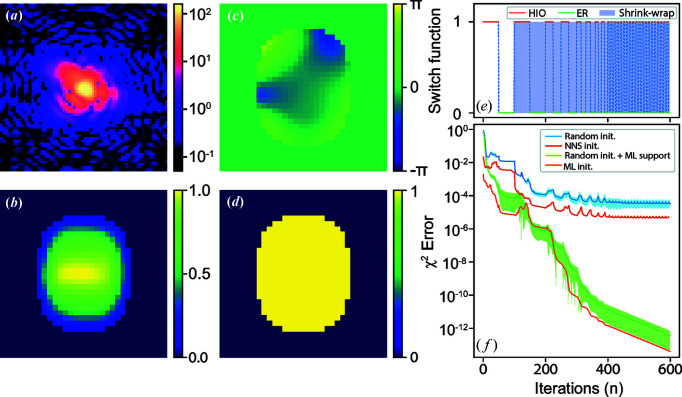
Comparison of the new CNN model with the classical iteration method. (*a*) A representative simulated 2D diffraction pattern from the testing data. (*b*), (*c*) Predicted amplitude and phase of the particle. (*d*) Support obtained by the binarization of the predicted amplitude. (*e*) The algorithm switch schedule for the iteration method. (*f*) The performance of the iterative phase-retrieval method with different initial guesses.

**Figure 6:**
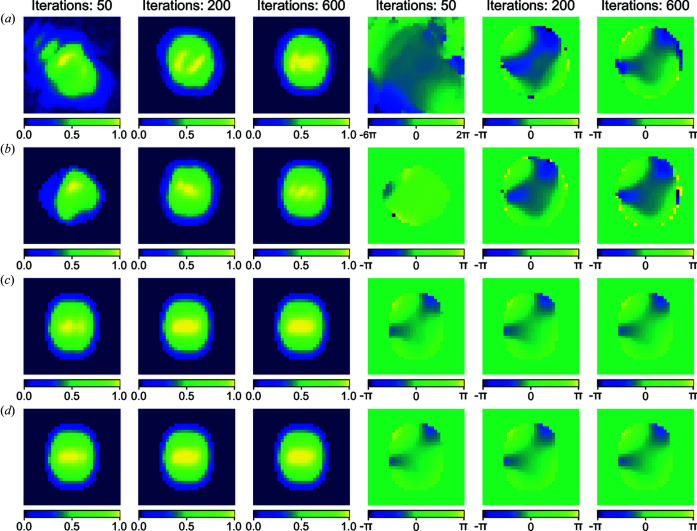
Representative reconstructed images *versus* iteration number at the different stages of iterative phase retrieval by using different initial starting methods. (*a*) Random initialization. (*b*) Using the NNS results as initialization. (*c*) Using the support from the machine-learning model and random phase as initialization. (*d*) Using the support and phase from the machine-learning model as initialization. The first three images in each row are the amplitudes of the particle and the rest are the corresponding phases. The corresponding iteration numbers of each of the images are given at the top of each column. When plotting the phase images of the particle, a 2D phase-unwrapping algorithm (see Appendix *C* [App appc] for details) is applied to make the obtained phase continually change.

We compare the outcomes of four different initializations of the iterative phase-retrieval method: (i) random phase and a rectangle support; (ii) random phase and the support from the CNN model; (iii) the phase and support from the CNN model; and (iv) the phase and support obtained from the NNS method. Fig. 5 ▸(*a*) shows one of the coherent X-ray diffraction patterns from the testing dataset with a strong phase inside the particle. The strong phase is evident in the broken centrosymmetry of the diffraction pattern. The trained CNN model yields the reconstructed amplitude and phase of the particle shown in Figs. 5 ▸(*b*) and 5 ▸(*c*). The corresponding estimated χ^2^ error for the modulus of the coherent X-ray diffraction pattern intensity is 0.015, and it can be seen that the CNN model gives an excellent-looking reconstructed result. The obtained CNN result was then used to define a support, shown in Fig. 5 ▸(*d*), by binarization of the predicted amplitude. Iterative phase-retrieval methods following the algorithm switch schedule indicated in Fig. 5 ▸(*e*) (see Appendix *B* [App appb] for details) were then applied using the different initial guesses, Fig. 5 ▸(*f*) shows the corresponding results in the conventional format of χ^2^ error *versus* iteration number. Owing to the initial phase being randomly generated for the methods of (i) random phase and a rectangle support and (ii) random phase and the support from the CNN model, the corresponding reconstructions for both methods were repeated 300 times independently. As presented in Fig. 5 ▸(*f*), the blue and green lines show the corresponding averaged χ^2^ error separately and the shaded areas indicate the error bars corresponding to their standard deviation. Furthermore, we also use the results (*i.e.* the amplitude and phase of the searched particle) from the NNS method as an initial guess for the iterative phase-retrieval method. The optimized amplitude and phase of the searched particle from the NNS method are presented in Fig. S1 of the Supporting information and the dependence on the size of the searched database is shown in Fig. S2. The corresponding χ^2^ error *versus* iteration number is also shown in Fig. 5 ▸(*f*), as marked by the red line. As seen in Fig. 5 ▸(*f*), with the CNN model initialization, the starting χ^2^ error was significantly lower than that of the random initialization or the initialization based on the NNS method. The final reconstruction was significantly better than what the CNN model and iterative method can achieve alone. This shows that machine learning with iterative refinement is an excellent combination for dramatic enhancement in reconstruction quality. The learned-phase initialization converged slightly faster than random phase, but the final result was not very different, while the use of the learned support made a large difference. Fig. 6 ▸ presents the evolution of these reconstructed images *versus* the iteration number with the different initial methods. It can be seen in Fig. 6 ▸ that the combination of the machine-learning model and iterative phase-retrieval converges much faster than the random initial guess or initialization from the NNS method.

**Figure 7:**
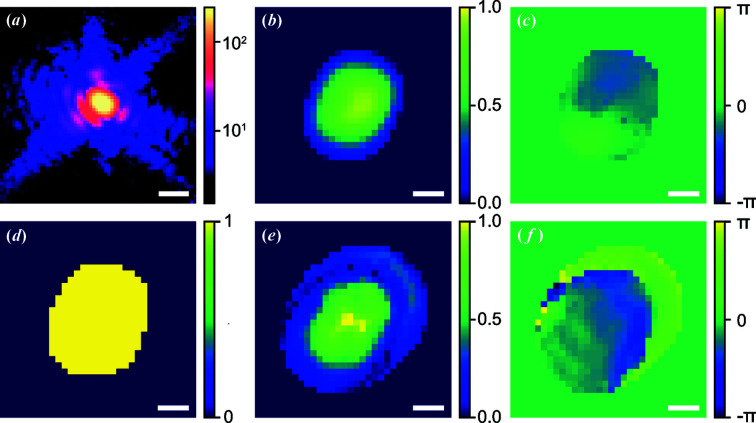
The performance of the CNN model with measured experimental data. (*a*) The central slice of a BTO single-particle diffraction pattern. Furthermore, reconstructed (*b*) amplitude and (*c*) phase of the BTO particle using the CNN model. (*d*) Support for the iterative phase-retrieval method, obtained by binarization of the output amplitude from the CNN model. Moreover, reconstructed (*e*) amplitude and (*f*) phase of the BTO particle using the combination of the iterative phase-retrieval method and the CNN model. Here, the scale bar is 50 µm^−1^ for (*a*). For (*b*)–(*f*), all scale bars are 100 nm.

Since the inside complex structure of a new particle is usually unknown, this combination approach becomes vital once the CNN model fails to give a decent result. This will often be expected for experimental data, when little knowledge of the structure is available for building a training dataset. To demonstrate this, Fig. 7 ▸(*a*) shows one representative experimental coherent X-ray diffraction pattern of an ∼200 nm diameter BaTiO_3_ (BTO) nanoparticle, measured at beamline 34-ID-C of the Advanced Photon Source using methods reported by Harder & Robinson (2013 ▸). By using the CNN model, the corresponding prediction is shown in Figs. 7 ▸(*b*)–7 ▸(*d*). The estimated χ^2^ error is 0.7 for the modulus of the coherent X-ray diffraction pattern intensity which shows that the current model has a poor performance for the given experimental data. This is attributed to the range of models used in the training data being far from the (unknown) structure of the particle. However, with the proposed combination of the CNN model results and the iteration method, Figs. 7 ▸(*e*) and 7 ▸(*f*) show a significantly better reconstruction result with a corresponding χ^2^ error reduced to 0.002. The iterative calculation used the CNN-generated support in Fig. 7 ▸(*d*) and utilized the ‘shrink wrap’ refinement shown in the work of Marchesini *et al.* (2003 ▸), which allowed it to both increase and decrease in size.

Based on these results, it can be concluded that our CNN method has great potential for studies to be performed in regimes of asymmetric data previously untested owing to the need to solve for a complex density function. Moreover, the combination of the CNN model with the classical iterative method can further improve the accuracy of the obtained results.

## 3. Conclusions   

In this article, we have put forward a comprehensive machine-learning model for the single-particle-imaging problem, where single shots of coherent X-ray diffraction patterns from a strong-phase object are recorded at an XFEL. Our CNN method shows high accuracy and speed for reconstructing the amplitude and phase information from the diffraction modulus in reciprocal space. We tested a superellipsoid particle with five degrees of freedom in its shape, three in its orientation and three in its phase structure. Crudely estimating ten possible settings for each degree of freedom yields 10^11^ possible structures. Our results show that the machine-learning approach gets much closer to phasing a novel structure than a simple search of the 150 000 structures used for training the CNN. The proposed machine-learning model can be used for real-time imaging at the high data-streaming rate of current XFEL sources. Furthermore, we also showed that using machine-learning results as a starting point can provide significant improvement of the accuracy of traditional iterative phase-retrieval methods. Since the machine-learning model learns directly from simulated diffraction data, this is important when meeting new experimental data. Currently, we are restricted to low-resolution 2D data, which takes more than a day of computer time to perform the training. Especially when extended to 3D, we believe our results have a very broad application in this and related research fields.

## Supplementary Material

Supporting information. DOI: 10.1107/S2052252520013780/cw5029sup1.pdf

## Appendix A

### A1. Data and CNN-model training

The 2D diffraction patterns were generated by taking the central slice of the 3D diffraction data, which is the Fourier transform of the generated complex-valued 3D array created from the particle’s amplitudes and phases. Only the amplitude of the computed diffraction patterns was retained for both training and testing of the CNN model. The generated training dataset contains a wide variety of amplitude and phase states, including some complex states with poor definition. For the loss *l*, here we propose a loss function that can constrain the real and reciprocal space at the same time:

where

and

*L*_1_(*A*) is the loss for the amplitude of the particle in real space and *L*_2_(ϕ) is the loss for the phase in real space, where *L*_2_(*x*) = *L*_1_(*x*).  is the loss for the X-ray diffraction intensity in reciprocal space, which is used to constrain the relation of the predicted amplitude and phase from the machine-learning model in reciprocal space. In the above equations, the subscript p denotes the predicted results from the machine-learning model and the subscript g denotes the corresponding ground truth. Additionally, in function *L*_3_(*x*), the second part is the Pearson correlation coefficient. Here, λ_1_ = 1, λ_2_ = 1 and λ_3_ = 4. The proposed CNN model was implemented based on the *Pytorch* platform (Paszke *et al.*, 2019 ▸) using *Python*. We adopted the stochastic gradient-descent optimizer with a learning rate of 0.01 to optimize the weights and biases, and to achieve a better convergence. After every ten epochs, the learning rate was reduced by a factor of 0.95. In our study, the size of the input coherent X-ray diffraction pattern was 64 × 64 pixels. The training took ∼12 h on a computer with 24 GB of RAM and an NVIDIA Quadro P100 GPU for 200 epochs. The Python codes used in this work will be made available to readers upon request to the authors.

## Appendix B

### b1. Phase-retrieval method

To perform phase retrieval, the simulated 2D diffraction was used as input to an iterative phase-retrieval scheme similar to that described by Robinson & Harder (2009 ▸), where the algorithm was switched between error reduction and hybrid input–output. 600 iterations were performed by using this phase retrieval. After 100 iterations, the shrink-wrap method of Marchesini *et al.* (2003 ▸) was used in real space to dynamically update the support every ten iterations. When using the shrink-wrap method to update the support, the size of the support is allowed to increase or decrease. In fact, there are two supports used during the shrink-wrap method in our code. Support 1 is dynamically adjusted to find the profile of the particle. Support 2 is just a fixed square support, which is an upper size limit used to confine Support 1. During the iterations, the χ^2^ error is used to estimate the quality of the reconstruction results, given by

where *I*_p_ is the reconstructed X-ray diffraction intensity and *I*_g_ is the true or experimental diffraction intensity.

## Appendix C

### c1. 2D phase-unwrapping method

The 2D phase-unwrapping algorithm is based on the method proposed by Herráez *et al.* (2002 ▸), which utilizes an ‘unwrapping path’ to solve the discontinuity of the phase obtained from a complex-valued object. First, the edge between each pair of neighbouring pixels is calculated based on their amplitude and phase. Then, based on these edge results, the pixels are divided into different groups. Finally, each of the different groups is offset by a multiple of 2π to make the phase of the input image change continually.
