# Supplementary material for: Complex imaging of phase domains by deep neural networks
Source: IUCrJ. 2021 Jan 1;8(Pt 1):12–21. doi: 10.1107/S2052252520013780 (PMC7792998; doi:10.1107/S2052252520013780)
Supplement: Supplementary file 1 [file m-08-00012-sup1.pdf]

IUCrJ

Volume 8 (2021)

Supporting information for article:

Complex imaging of phase domains by deep neural networks

Longlong Wu, Pavol Juhas, Shinjae Yoo and Ian Robinson

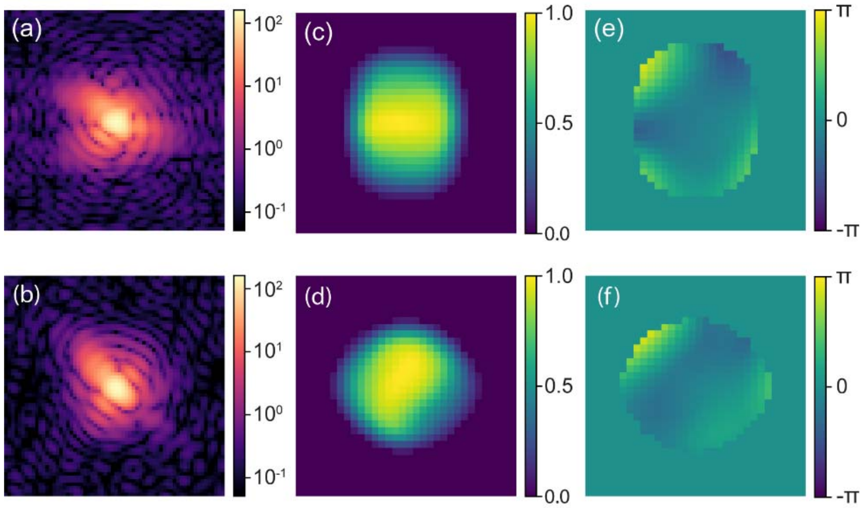

Figure S1 The comparison between the optimized result obtained by the nearest-neighbour method and its ground truth. (a) The input diffraction pattern for the nearest-neighbour method and (b) the diffraction pattern of the searched particle. (c) The ground truth for the amplitude the particle and (d) the corresponding amplitude of the searched particle. (e) The ground truth for the phase of the particle and (f) the corresponding phase distribution of the searched particle.

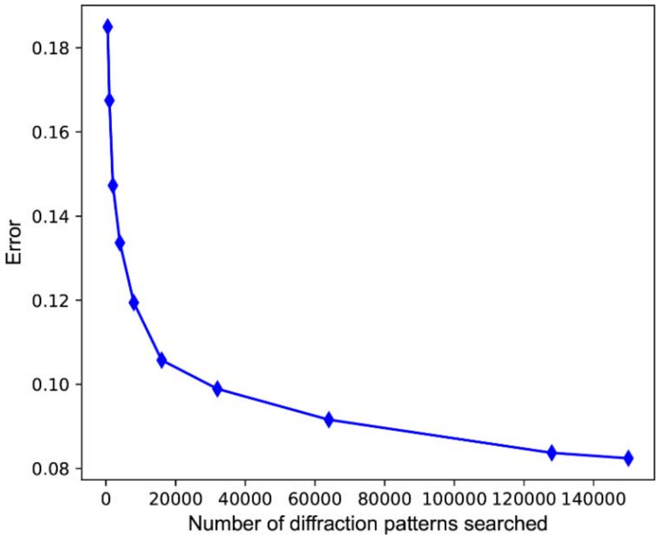

Figure S2 The error from nearest-neighbour method as a function of the size of the its database. To obtain a statistical relevant result in each case, the error is calculated by an average over 300 different diffraction patterns. It can be seen that the error decreases roughly as expected with the number of data. However, even when the size of the dataset reaches 150,000, the error is only ~0.08, much greater than the 0.02 error given by given by Machine Learning model with the same number of training data size.
